# CL-PMI: A Precursor MicroRNA Identification Method Based on Convolutional and Long Short-Term Memory Networks

**DOI:** 10.3389/fgene.2019.00967

**Published:** 2019-10-11

**Authors:** Huiqing Wang, Yue Ma, Chunlin Dong, Chun Li, Jingjing Wang, Dan Liu

**Affiliations:** ^1^College of Information and Computer, Taiyuan University of Technology, Taiyuan, China; ^2^Dryland Agriculture Research Center, Shanxi Academy of Agricultural Sciences, Taiyuan, China

**Keywords:** pre-miRNA identification, long short-term memory network, convolutional neural network, deep learning, imbalanced learning

## Abstract

MicroRNAs (miRNAs) are the major class of gene-regulating molecules that bind mRNAs. They function mainly as translational repressors in mammals. Therefore, how to identify miRNAs is one of the most important problems in medical treatment. Many known pre-miRNAs have a hairpin ring structure containing more structural features, and it is difficult to identify mature miRNAs because of their short length. Therefore, most research focuses on the identification of pre-miRNAs. Most computational models rely on manual feature extraction to identify pre-miRNAs and do not consider the sequential and spatial characteristics of pre-miRNAs, resulting in a loss of information. As the number of unidentified pre-miRNAs is far greater than that of known pre-miRNAs, there is a dataset imbalance problem, which leads to a degradation of the performance of pre-miRNA identification methods. In order to overcome the limitations of existing methods, we propose a pre-miRNA identification algorithm based on a cascaded CNN-LSTM framework, called CL-PMI. We used a convolutional neural network to automatically extract features and obtain pre-miRNA spatial information. We also employed long short-term memory (LSTM) to capture time characteristics of pre-miRNAs and improve attention mechanisms for long-term dependence modeling. Focal loss was used to improve the dataset imbalance. Compared with existing methods, CL-PMI achieved better performance on all datasets. The results demonstrate that this method can effectively identify pre-miRNAs by simultaneously considering their spatial and sequential information, as well as dealing with imbalance in the datasets.

## Introduction

MicroRNAs (miRNAs) are ribonucleic acid molecules of about 21–23 nucleotides that are widely found in microorganisms, viruses (Pfeffer et al., 2004), and plants (Jones-Rhoades et al. 2006). They are known to regulate thousands of human genes that account for more than one-third of the genomic coding region (Bentwich et al., 2005). miRNAs also have important roles in the pathogenesis and treatment of cancer (Wang et al., 2010; Jansson and Lund, 2012; Tüfekci, et al., 2014; Zhu et al., 2014). A study has shown that 50% of miRNAs frequently appear in tumor-associated gene regions or fragile sites such as homozygous deletion regions, heterozygous deletion regions, amplification regions, and breakpoint regions, as well as in proximity to tumor suppressor genes and the locations of oncogenes, indicating a correlation between the localization of miRNAs on human chromosomes and tumorigenesis (Calin et al., 2004). In addition, miRNAs are potential targets for disease markers and therapeutic drugs (Schmidt, 2014), for instance, they guide the RNA-induced silencing complex to degrade or inhibit mRNA translation by pairing with bases of the target gene mRNA, thereby regulating protein expression at the post-transcriptional level (research has shown that miRNAs can also regulate gene expression at the transcriptional level). Therefore, how to identify miRNAs is a key question with implications for medical treatment. miRNAs exist in many forms; the most primitive of these is primary miRNA, which becomes precursor miRNA (pre-miRNA) after single processing. The pre-miRNA is digested by Dicer to form a mature miRNA (Agarwal et al., 2010). It is difficult to identify mature miRNAs owing to their short length; thus, most previous studies have focused on identifying pre-miRNAs.

pre-miRNA identification is a binary classification task requiring the input of a given set to be classified into two groups, producing precursors and non-precursors as the output. A large number of computational methods for identifying miRNAs have been proposed; these can be divided into experimental cloning and computer simulation prediction methods (Bartel, 2004; Jones-Rhoades et al., 2006). Experimental methods are recognized as the gold standard for miRNA identification; however, it is impossible to discover all miRNAs through experimental cloning because of the small number of discoveries and the specific development time or specific tissue expression. Computer simulation methods can be used to obtain reliable predictions and reduce the cost of research and production, and they have been proven to effectively detect miRNAs expressed in specific tissues (Bartel, 2004). Among the available computer simulation prediction methods for the identification of miRNAs, rule-based methods (Mathelier and Carbone, 2010) and machine learning methods have been widely applied. These include microPred (Batuwita and Palade, 2009b), triplet-SVM (Xue et al., 2005), and miRBoost (Tempel et al., 2015), which use different numbers of human and cross-species manual features to identify miRNAs as inputs to a support vector machine(SVM); and MiPred (Jiang et al., 2007), which selects a set of mixed features, including the minimum free energy (MFE), the local contiguous triplet structure composition, dinucleotide shuffling, and the P-values of randomization tests, to construct a random forest classifier to identify miRNAs. The context-sensitive hidden Markov model (CSHMM) method (Agarwal et al., 2010) predicts miRNAs by filtering the human dataset; whereas M0iRANN (Rahman et al., 2012), DP-miRNA (Thomas et al., 2017), and BP (Jiang et al., 2016) extracted 98 features as inputs to their neural networks. These methods use hand-crafted features as inputs to the model, including pre-miRNA structural and folding energy information such as dinucleotide and trinucleotide pair frequency, loop and sequence length, MFE, and melting temperature. Manual extraction of features often requires careful design based on the characteristics of the data; this, combined with reliance on the database, weakens the generalization ability of the model.

Many deep learning methods can automatically learn the representation of features from the data. For instance, deepMiRGene (Park et al., 2017) uses long short-term memory (LSTM) to automatically extract features and process time-dependent problems in a sequence. Do et al. (2018) introduced a convolutional neural network (CNN) to automatically extract features to identify miRNAs. Lee et al. (2016) used an automatic encoder based on a deep recurrent neural network (RNN) to determine the interaction of miRNA sequences for miRNA target prediction. All of these methods involve automatic extraction of features. However, most of the information is both spatial and sequential. The miRNA spatial structure contains miRNA functional information, as the base sequence of the miRNA affects the normal regulation of miRNA molecules. Each of these methods focuses on either time or spatial information.

In recent years, researchers have explored how to use CNN and RNN tools to construct various CNN-RNN frameworks, which can be divided into unified and cascaded combinations (Pinheiro and Collobert, 2014; Donahue et al., 2015; Vinyals et al., 2015; Zuo et al., 2015; Wang et al., 2016; You et al., 2016). In these, the cascaded framework processes the CNN and the RNN, respectively, and the RNN takes the output of the CNN as its input and returns continuous predictions at different time steps. Such cascaded frameworks can handle various tasks. For example, Pinheiro et al. replaced an RNN with LSTM to solve image subtitle tasks using CNN-RNN (Pinheiro and Collobert, 2014). The model trained the CNN to identify objects in video frames and classify them, then used the output of the CNN as input to the LSTM, creating an “instant” description for each video clip. Quang et al. proposed the DanQ CNN/LSTM combination model (Quang and Xie, 2016), which models the nature and function of introns, using a convolutional layer to capture the regulatory motif while the recursive layer captures the inter-model long-term dependencies, and demonstrated its ability to learn a regulatory grammar to improve forecasting. Compared with other models, DanQ showed great improvements with respect to many metrics. Pan et al. used CNN to learn abstract features and used bidirectional LSTM (BLSTM) to capture possible long-range dependencies between binding sequences and structural motifs recognized by CNN. In this way, they predicted sequence and structural binding preferences of RNA-protein complexes (Pan et al., 2018). These successful applications demonstrate that the ability to focus on both sequential and spatial characteristics yields better classification results.

However, these models cannot focus simultaneously on the sequential and spatial characteristics of pre-miRNAs, because they use a single neural network. They also disregard the information carried by the sequence owing to their focus on the secondary structure. Therefore, we proposed a pre-miRNA identification method based on a cascaded CNN-LSTM framework, called CL-PMI. First, CL-PMI uses the CNN to automatically learn the characteristics of the sequence and the secondary structure from the input, thereby obtaining a spatial feature representation of the pre-miRNAs. Then, a deep RNN with LSTM is used to capture pre-miRNA long-term dependence information from the effective features of CNN learning. Finally, the CL-PMI uses a fully connected layer to identify pre-miRNAs. Our approach involves a series of nonlinear transformations on data, performed in a data-driven manner, and uses hybrid neural networks to learn the complex abstract sequential and spatial features of data with the automatic extraction of features.

## Materials and Methods

Most existing miRNA identification algorithms manually extract features, which requires strong expertise in the field and thus inevitably limits their universality. These algorithms focus on either the sequential characteristics of the miRNA or its spatial characteristics, but not both.

**Figure 1:**
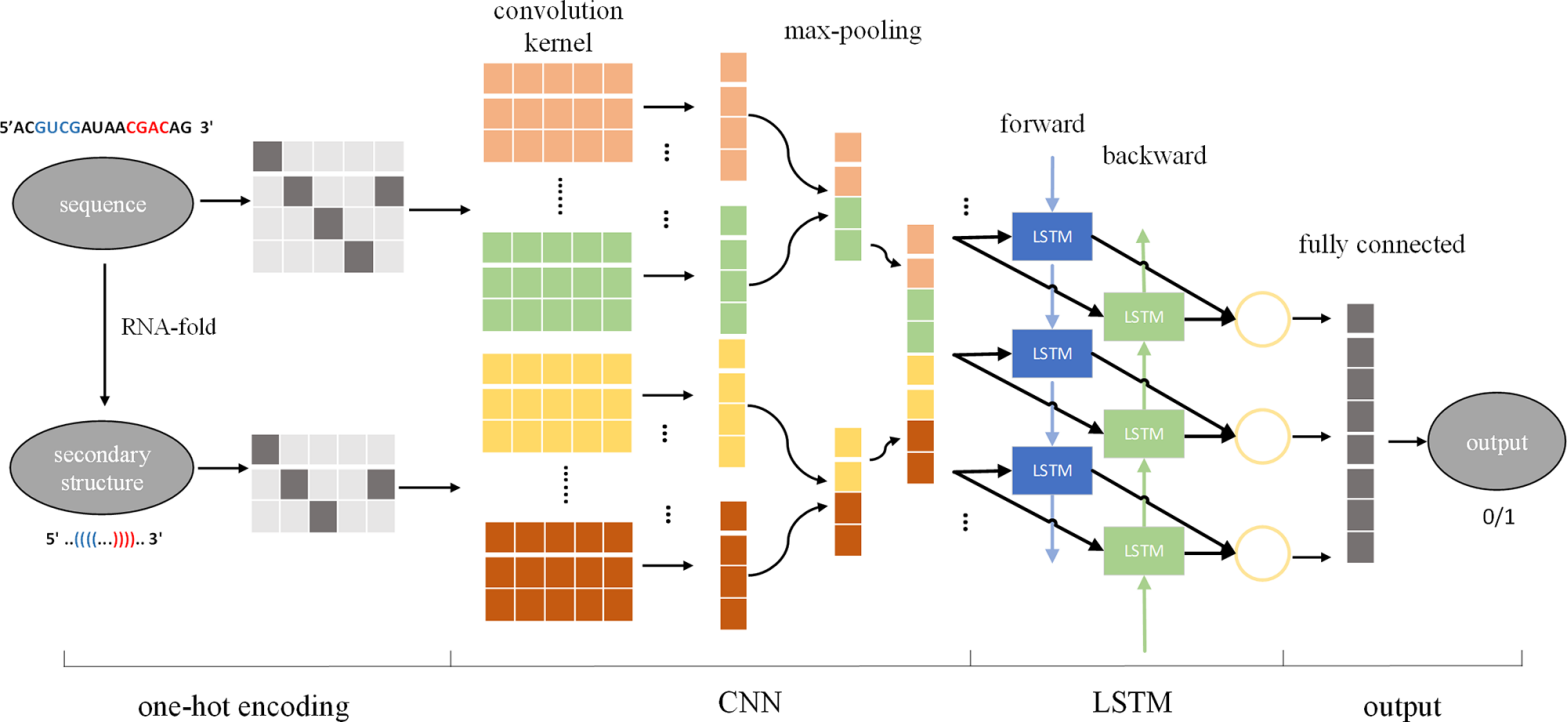
Overview of proposed CL-PMI methodology; the sequence and the secondary structure are first encoded as four-dimensional and three-dimensional matrices, respectively. The convolutional layer acts as a scanner across the input matrix to extract features, and the max-pooling layer reduces the data dimension and retains significant features. Then two CNNs are integrated as inputs to BLSTM, and the combined information is used to learn the long-term dependence of the pre-miRNA. Finally, the fully connected layer performs a sigmoidal nonlinear transformation on the output of BLSTM to identify the pre-miRNA.

Using the input sequence and secondary structure to design the size, number, and sliding step size of the convolution kernel, CNN can be used to automatically extract features from the input, effectively solving the problem of manual extraction of features. In the cascaded CNN-RNN framework, the CNN describes the state of a certain space, and the RNN connects the spatial states together to form a time concept, thus enabling the model to consider spatial and sequential characteristics at the same time. Therefore, we introduced a cascaded CNN-LSTM framework to identify pre-miRNAs, called CL-PMI, which consists of a CNN layer, an LSTM layer, and a fully connected layer (FC). In this framework, we first use one-hot encoding to process the pre-miRNA sequence and its corresponding secondary structure, using encoded pre-miRNAs as the input to the CNN. The CNN automatically extracts pre-miRNAs spatial correlation features; then, LSTM takes the effective features of CNN learning as inputs and uses the three gating units to capture the long-term dependencies of the pre-miRNAs. Finally, the fully connected layer combines spatial information and sequential information for robust classification. **Figure 1** shows an overview of the CL-PMI framework.

### Encoding Sequence and Structure

**Figure 2:**
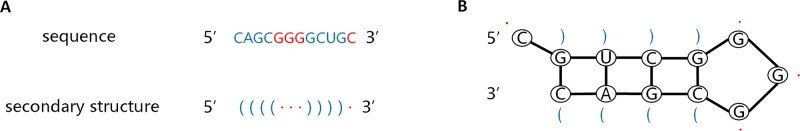
Sequence of pre-miRNA and corresponding secondary structures predicted by RNAfold. **(A)** Sequence of pre-miRNA and secondary structures described by dot-bracket notation. **(B)** Secondary structure of the given sequence.

The information carried by the sequence and secondary structure of the pre-miRNA plays an important part in the identification process. The pre-miRNA sequence is a non-coding single-stranded RNA molecule of approximately 22 nucleotides. The secondary structure is double-stranded owing to base pairing interactions. The stem ring and hairpin structures resulting from these interactions, as shown in **Figure 2B**, are the most prominent features of pre-miRNAs. The left side of the stem is the forward chain (5'→3'), and the right is the reverse strand (3'→5'), complementary base matches between these strands result in formation of a helix. Dot bracket notation (DBN) is a widely used method for describing secondary structures. As shown in **Figure 2A**, DBN uses paired parentheses to indicate complementary pairing of bases and continuous dot numbers to indicate stem-loop structures. The pre-miRNA secondary structure is one of the inputs of CL-PMI, which is obtained by calculating the MFE of the pre-miRNA sequence with the RNAfold tool (Hofacker, 2003).

In order to capture more pre-miRNA information, we considered the sequence information and corresponding secondary structure information simultaneously. Each pre-miRNA sequence consists of four nucleotide types {A, C, G, U}, and the secondary structure has three “(”, “.”, “)” observable states. We used a one-hot encoding scheme to convert the nucleotides at each position of the pre-miRNA sequence into four-dimensional vectors, and the observable state of each position of the secondary structure was converted into a three-dimensional vector; these vectors were used as the inputs to the CNN. For example, let *S* *_seq_* be a pre-miRNA sequence and *S* *_str_* be the secondary structure corresponding to *S* *_seq_*, where *S* *_seq_* = {*A,C,G,U,U*} and *S* *_str_* = {(,·,·,·,)}; then, *S* *_seq_* is encoded as a four-dimensional binary tuple vector and *S* *_str_* is encoded as a three-dimensional binary tuple vector:

$$S_{\mathrm{seq}}:[1,0,0,0],[0,1,0,0],[0,0,1,0],[0,0,0,1],[0,0,0,1]$$

$$S_{\mathrm{str}}\;:[1,0,0],[0,1,0],[0,1,0],[0,1,0],[0,0,1]$$

### Convolutional Neural Network

A pre-miRNA sequence contains frequency-dependent features of two or three adjacent nucleotide and aggregated dinucleotide frequencies. The secondary structure of pre-miRNA involves different thermodynamic stability spectra of the pre-miRNA and other features, such as adjusted base pair distance, structure entropy, melting temperature, loop length, and positional entropy, which estimates the structural volatility of the secondary structure. As the pre-miRNA sequence and the secondary structure carry different information characteristics, we used CNN to train two different branches for the sequence and the secondary structure and to learn the subsequence features from the two types of input information.

Each CNN branch consisted of a convolutional layer, a rectified linear unit (ReLU), and a max-pooling layer that together extracted sequence and secondary structure features from the input. We selected the max-pooling layer to subsample the output of the convolutional layer. There are two advantages to using max-pooling. First, it reduces the offset of estimated mean caused by convolutional layer parameter errors. Second, it removes redundant information carried by the feature map, reduces parameters, and prevents overfitting. The convolutional layer extracts the features of the input data and abstracts the implicit associations in the original data through the convolution kernel matrix. In principle, convolution is a mathematical operation of point-multiplication summation of two matrices, the input data matrix and the convolution kernel (filter or feature matrix). The results obtained are expressed as specific local features extracted from the pre-miRNA. After convolution, we applied a rectifying linear unit to sparsify the output of the convolutional layer, then output the region vector obtained by the pooling layer to the LSTM layer.

For the sequence, the input matrix length was denoted by *b*, and the convolutional layer included *N* *_filter_* filters, each of length *k*. Each sliding window range was *s*=1 to *b*-*k*+1. A sliding filter and point multiplication were used to obtain a feature map of size *N* *_filter_*×(b-k+1). The convolved feature map, *Z*, can be represented as follows:

(1) $$Z=f_{\mathrm{conv}}(X)$$

(2) $$Z_{s,i}=\sum_{j=1}^{N_{f}}\sum W_{i,j,r}X_{s+r-1,j}+B_{i}$$

where *Z* *_s,i_* represents the feature map generated by the *s*th sliding neighborhood window and the *i*th filter; *X* is the input sample, of size *N* *_in_*×*b*, i∈{1,…,*N* *_filter_*}; W is the weight, of size *N* *_filter_*×*N* *_in_*×*k*; and B is the bias value, of size *N* *_filter_*×1. These are the trainable parameters of the convolution layer.

Next, we applied a ReLU, an activation function that keeps the convolutional layer positively matched and eliminates negative matches:

(3) $$f_{\mathrm{relu}}(Z)=\mathrm{relu}(Z)=\max(0,Z)$$

In order to reduce the parameters and learn translational invariant features, we used max-pooling on the output of the convolution. Max-pooling preserves only the maximum output of each filter in each step to reduce the output size of the convolution layer; it was applied to the output of convolution Z of size *N* *_filter_*×*s*, where *s*=*b-k*+1. In the case where the size of the pool was *m*, we obtained an output *V*

(4) $$V=f_{\mathrm{maxpool}}(Z)$$

(5) $$V_{i,p}=\begin{array}{c} m\\ \max\\ j=1 \end{array}Z_{i,m(s-1)+j}$$

where $s\in \left\{1,\ldots ,\left\lfloor \frac{s}{m}\right\rfloor \right\}$, *i* ∈{1,…,*N* *_filter_*} and the size of V is $N_{\mathrm{filter}}\times \left\lfloor \frac{s}{m}\right\rfloor$. Analogous definitions also hold for secondary structure.

Before entering the next layer, the sequence and the secondary structure were concatenated into a single output. The next LSTM layer and the fully connected layer worked together on the merged sequence and the structural layer.

### Long Short-Term Memory Network

**Figure 3:**
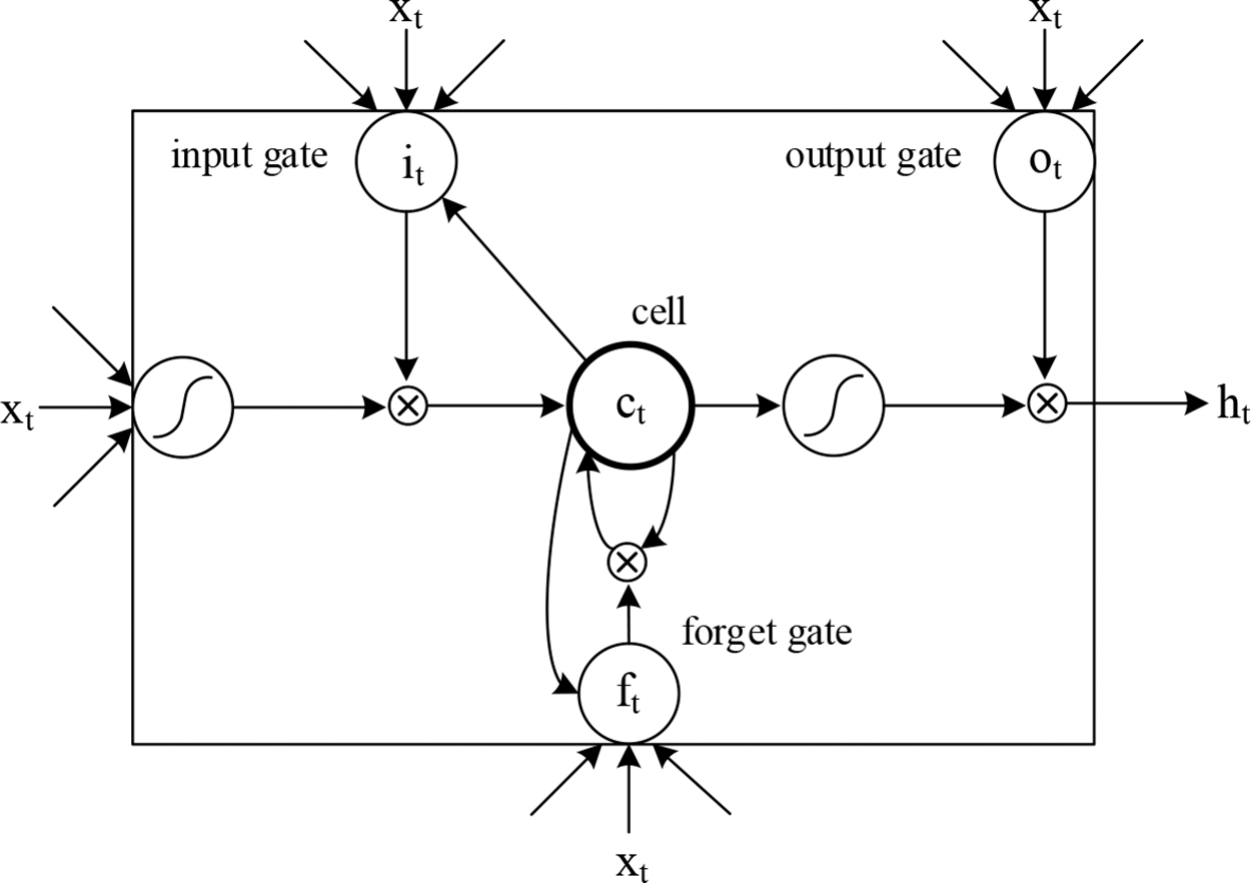
Internal structure of the LSTM cell.

We introduced LSTM (Hochreiter and Schmidhuber, 1997) to identify the combined information of the sequence and secondary structures, allowing us to use long-term dependency information to aid current predictions. An LSTM cell has an internal mechanism called a gate that regulates the flow of information. Three gate units are shown in **Figure 3**. When the LSTM cell scans each element of the input sequence, it first selectively discards the information in the cell state using the “forget” gate. The input gate records new information into the cell state and then updates the current state value. Finally, the output gates determine which values should be output. As standard LSTM often ignores the future context of the pre-miRNA when processing the sequence, a bidirectional LSTM (Graves et al., 2005) is used to solve this problem. Its main goal is to increase the information available to the RNN, including the history and future data of an input using time series data. It scans the outputs of the CNN from two directions, along and against the timing direction. The outputs for each direction are connected for subsequent classification. The calculation process of the LSTM cell at the time step *t* is as follows:

(6) $$i_{t}=\;\sigma (W_{\mathrm{xi}}x_{t}+W_{\mathrm{hi}}h_{t-1}+W_{\mathrm{ci}}c_{t-1}+b_{i})$$

(7) $$f_{t}=\;\sigma (W_{\mathrm{xf}}x_{t}+W_{\mathrm{hf}}h_{t-1}+W_{\mathrm{cf}}c_{t-1}+b_{f})$$

(8) $$c_{t}=\;f_{t}c_{t-1}+i_{t}\tanh(W_{\mathrm{xc}}x_{t}+W_{\mathrm{hc}}h_{t-1}+b_{c})$$

(9) $$o_{t}=\;\sigma (W_{\mathrm{xo}}x_{t}+W_{\mathrm{ho}}h_{t-1}+W_{\mathrm{co}}c_{t}+b_{o})$$

(10) $$h_{t}=o_{t}\tanh(c_{t})$$

where *f* *_t_*, *i* *_t_*, *c* *_t_*, *o* *_t_* represent the forget gate, the input gate, the cell activation vector, and the output gate, respectively; *X*, *h*, and *c* represent input vectors, hidden states, and memory cells, respectively; *W* and *b* are the weights and bias, that is, the model parameters to be trained; and *σ* is the sigmoid function:

(11) $$\sigma (x)=1/(1+e^{-x})$$

### Addressing Potential Overfitting

Overfitting is a very common problem in deep learning and may result from the model lacking control during the learning process. An overfitting model will not perform well in data identification. In the current work, the model would not be able to identify pre-miRNA correctly if overfitting were to occur and would have poor generalization ability. In order to reduce this risk, we used batch normalization, dropout, and L2 regularization to prevent or mitigate overfitting.

Batch standardization (Ioffe and Szegedy, 2015) normalizes the output of neurons in each training batch so that the output obeys a normal distribution with 0 as the mean and 1 as the standard deviation, thus avoiding the problem of internal covariate migration. In the case where the *i*th batch contains 100 samples, the particular neuron produces outputs *N* *_i_*_,1_, … ,*N* *_i_*_,100_, then standardizes it in batches:

(12) $$\frac{N_{i,1}-{\bar{N}}_{i}}{\sigma_{i}},\ldots ,\frac{N_{i,100}-{\bar{N}}_{i}}{\sigma_{i}}$$

where $\sigma_{i}^{2}=\frac{1}{n-1}\sum_{j=1}^{100}{\left(N_{i,j}-\;{\bar{N}}_{i}\right)}^{2}\;\mathrm{and}\;\;{\bar{N}}_{i}=\frac{1}{n}\sum_{i,j}^{100}N_{j=1}$ are the sample variance and mean, respectively. Batch normalization makes the feature scales consistent in all dimensions of the data to avoid excessive concentration of some dimensional data, thus alleviating gradient disappearance and overfitting. By reducing the dependence of the gradient on the parameters or their initial scales, the network can be trained with a high learning rate to accelerate network convergence. Batch normalization can be seen as a means of regularization, which can improve the generalization ability of the model and optimize the model structure. We batch-standardized the output of the max-pooling layer, the LSTM layer, the fully connected layer, and the ReLU activation in the network. During the forecast period, the batch average and variance were replaced by the total mean and variance, which were calculated when all batches had been trained.

Dropout (Srivastava et al., 2014) refers to temporarily dropping the output of the neural network unit from the network according to a fixed probability *p* during the training of the deep learning network. In other words, the effects of these neurons on the downstream start-up are neglected in the forward propagation, and their weights are not updated in the backpropagation. This makes the network less sensitive to changes in the weight of a neuron, increasing generalization and reducing overfitting. For the max-pooling layer, the LSTM layer, and the fully connected layer, we applied a loss rate of *p=0.5* to the outputs. Note that dropout was only used during training. In the testing stage, dropout was not applied because a random output would affect the prediction.

Finally, we applied L2 regularization to the weight matrix of the fully connected layer, and punished the loss function by adding the square of the weight to the loss function. This reduced the complexity of the model, thereby reducing overfitting.

### Training

We implemented our neural network model using Python and Keras (Chollet, 2015). The model utilized a backpropagation algorithm to calculate the loss function value between the output and the label, before calculating its gradient relative to each neuron and updating the weight according to the gradient direction. We applied focal loss (Lin et al., 2017), a dynamically scaled cross-entropy loss function, to train the samples. Formally, focal loss is defined as

(13) $${\text{L}}_{\mathrm{FL}}={\left(1-p_{y}\right)}^{\gamma }L_{\mathrm{CE}}$$

(14) $$L_{\mathrm{CE}}=-\alpha_{y}\log p_{y}$$

(15) $$p_{y}=\{\begin{array}{cc} p & \mathrm{if}\;y=1\\ 1-p & \mathrm{otherwise} \end{array}$$

where *L* *_CE_* represents the cross-entropy of binary classification; y ∈ {+1,−1} specifies the label of the sample; p ∈ [0,1] is the model’s estimated probability for the class with label y = 1; α ∈ [0,1] is a weighting factor corresponding to class 1, and 1-α corresponds to class -1; γ is the focus parameter; and (1-*p* *_y_*)^γ^ is the modulation factor. In this work, we set γ to 2 and α to 0.25.

In the process of training a model, choosing a good optimizer not only accelerates the training of the model but also improves the experimental results. Kingma and Ba (2014) proposed the Adam optimizer, which combines the advantages of two algorithms, AdaGrad and RMSProp, to calculate the update step by considering the first moment estimation (the mean of the gradient) and the second moment estimation (the uncentered variance of the gradient). Furthermore, Adam is considered to be an optimizer with excellent performance and is the default choice. Therefore, we chose Adam as the optimizer while training CL-PMI, with the mini-batch size and learning rate set to 128 and 0.001 for all experiments. The details of the model parameters are shown in **Supplement A** Pseudo code can be obtained in **Supplement B**.

**Figure 4:**
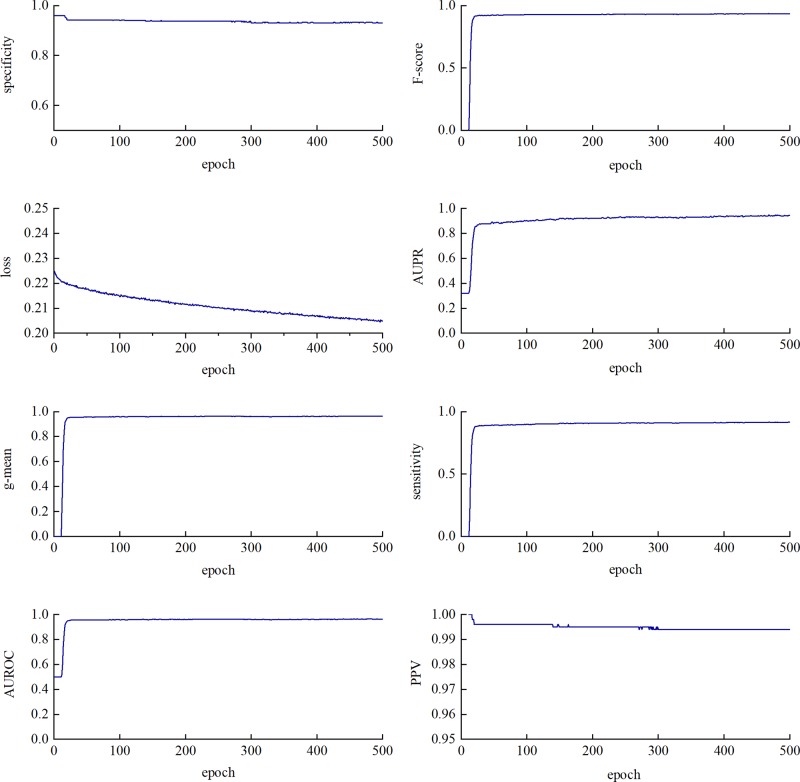
Training loss and seven evaluation metrics using test dataset with a varying epoch. The changes in metrics and loss functions tended to be stable after about 300 epochs, so we used a model that trained 300 epochs.

We performed a five-fold cross-validation on the training data to evaluate the classification performance of CL-PMI. As shown in **Figure 4**, SP, F-score, g-mean, and AUROC tend to be stable at about 20 epochs. SE and AUPR showed a slight upward trend between 20 and 300 epochs. The PPV fluctuated around 280 epochs and stabilized after 300 epochs. Loss has been in a gentle downward trend. Different indicators are stable in different epoch, in order to comprehensively consider all indicators, we stopped training after 300 epochs. Owing to the data imbalance, the prediction was biased towards the negative dataset in the early training stage, as reflected by the F-score and geometric mean (g-mean) remaining close to 1. However, the prediction was tuned and converged as learning progressed.

## Results and Discussion

Our method and the four comparison methods used the same datasets (Tempel et al., 2015; Park et al., 2017), *human*, *cross-species*, and *new*. Positive examples were retrieved from miRbase (Griffiths-Jones et al., 2006) (18th edition), and the negative examples were from NCBI (http://www.ncbi.nlm.nih.gov), NONCODE (Bu et al., 2011), fRNAdb (Kin et al., 2006), and snoRNA-LBME-db (Lestrade and Weber, 2006). Negative examples mainly included exonic regions from protein-coding genes and noncoding RNAs that were not miRNAs, such as tRNA, siRNA, snRNA, and snoRNA. To improve data quality and prevent overfitting, mis-annotated elements were discarded in these examples and redundant sequences were removed (Tempel et al., 2015). In addition, we obtained 690 positive samples from miRBase22, and obtained 8,246 negative examples from Xue (Xue et al., 2005) and Zou (Wei et al., 2014) as new22 dataset. For the human and cross-species datasets, we used 10% of the data as the test set and the remaining 90% to implement five-fold cross-validation for training and selecting the model. The new and new22 dataset were only used for testing. We predicted for new and new22 dataset using the model trained with cross-species dataset.

As shown in **Table 1**, the experiment involved a total of 3,230 positive examples and 23,934 negative examples. The human dataset contained 863 positive examples and 7,422 negative examples. The cross-species dataset consisted of 1,677 positive examples and 8,266 negative examples obtained from different species (e.g., mice, humans, and flies). The new dataset was obtained from miRBase versions 19 and 20, and consisted of 690 positive and 8,246 negative newly found examples.

**Table 1:** The number of sequences in the datasets used in this study.

	Cross-species	Human	New	New22
Positive examples	1,677	863	690	690
Negative examples	8,266	7,422	8,246	8,246

### Experimental Setup

For the human and cross-species datasets, we performed a five-fold cross-validation. We randomly selected 80% of the data to form the training set; the remaining 20% were used as the test set. The numbers of hidden nodes in the LSTM and FC layers were determined to be 20 and 256 by five-fold cross-validation. The mini-batch size and training epochs were set to 128 and 300, respectively.

For comparison, sensitivity (SE), specificity (SP), F-score, g-mean, positive predictive value (PPV), area under the precision-recall curve (AUPR), and area under the receiver operating characteristic (AUROC) were used to evaluate model performance. These metrics were calculated as follows:

(16) SE=TP/(TP+FN)

(17) SP=TN/(TN+FP)

(18) PPV=TP/(TP+FP)

(19) F−score=2TP/(2TP+FP+FN)

(20) g−mean=√SE⋅SP

where TN, TP, FN, and FP denote the number of true negatives, true positives, false negatives, and false positives, respectively. These formulas were based on the confusion matrix, with a decision threshold of 0.5.

### Validation and Test Performance Evaluation

Next, CL-PMI was applied to three datasets for pre-miRNA identification. In order to evaluate the performance of CL-PMI, we compared it with four existing pre-miRNA identification methods.

One of these methods, miRBoost (Tempel et al., 2015), is an ensemble method that extracts the appropriate features from 187 existing features and performs classification after training the data using the enhancement techniques of the SVM component. Another, microPred (Batuwita and Palade, 2009b), selects the most discriminative feature setting to train the SVM classifier using a filtering method, handles the class imbalance problem in the dataset, and uses cross-validation to evaluate classification performance. The SVM used in these two methods is a binary classification model, which can be defined as the linear classifier with the largest interval in the feature space. The learning strategy is to maximize the interval and finally transform into a solution to a convex quadratic programming problem. These two comparison methods are traditional machine learning methods.

Park et al. (2017) combined secondary structure with the pre-miRNA sequence to form a 16-dimensional matrix, then sent the results to the RNN to improve long-term dependency modeling. The greatest advantage of this approach is that it does not require hand-crafted features. Do et al. (2018) proposed a novel joint two-dimensional multi-channel method to identify pre-miRNAs, using the secondary structure encoded by the pairing matrix format as the input to the two-dimensional convolution network to achieve automatic feature extraction. These features were fed into fully connected layers for classification. These two comparison methods are deep learning methods.

Therefore, we used miRBoost, microPred, deepMiRGene, and DCNN as comparative experiments in this paper to evaluate the performance for pre-miRNA identification under the same datasets. The experimental results for the three datasets are described and discussed below.

**Tables 2** and **3** show the cross-validation and test performance for the human and cross-species data-sets, respectively. Cross-validation performance is shown in the top half of each table and test performance in the lower half.

**Table 2:** Results for the human dataset.

Methods\metrics	SE	SP	PPV	F-score	g-mean	AUROC	AUPR
miRBoost (CV)	0.803	0.988	0.887	0.843	0.891	−	−
microPred (CV)	0.763	**0.989**	0.888	0.820	0.869	0.974	0.890
deepMiRGene (CV)	0.799	0.988	0.885	0.839	0.888	0.984	0.915
DCNN fixed-sized (CV)	0.878	0.978	0.827	0.849	0.926	0.984	0.915
DCNN variable-sized (CV)	0.835	0.985	0.868	0.851	0.907	**0.985**	**0.922**
Proposed (CV)	**0.989**	0.935	**0.992**	**0.991**	**0.962**	0.962	0.854
miRBoost (test)	0.884	0.969	0.768	0.822	0.925	−	−
microPred (test)	0.779	0.988	0.882	0.827	0.877	0.980	0.892
deepMiRGene (test)	0.822	**0.992**	0.919	0.868	0.903	0.981	0.918
DCNN fixed-sized (test)	0.930	0.984	0.870	0.899	**0.957**	0.983	**0.946**
DCNN variable-sized (test)	0.884	0.991	0.916	0.899	0.936	**0.986**	0.934
Proposed (test)	**0.968**	0.895	**0.988**	**0.978**	0.931	0.972	0.807

Bold numbers are the highest scores in this catergory.

**Table 3:** Results for the cross-species dataset.

Methods\metrics	SE	SP	PPV	F-score	g-mean	AUROC	AUPR
miRBoost (CV)	0.861	0.977	0.884	0.872	0.917	−	−
microPred (CV)	0.825	0.975	0.875	0.848	0.897	0.970	0.873
deepMiRGene (CV)	0.886	**0.982**	0.911	0.898	0.933	**0.985**	0.927
DCNN fixed-sized (CV)	0.903	0.978	0.894	0.898	0.940	**0.985**	0.936
DCNN variable-sized (CV)	0.881	0.981	0.906	0.893	0.930	0.983	**0.936**
Proposed (CV)	**0.995**	0.950	**0.990**	**0.992**	**0.972**	0.972	0.933
miRBoost (test)	0.856	0.844	0.526	0.651	0.850	−	−
microPred (test)	0.814	0.985	0.919	0.863	0.896	0.963	0.906
deepMiRGene (test)	0.900	0.983	0.913	0.906	0.940	0.984	**0.955**
DCNN fixed-sized (test)	0.904	0.982	0.910	0.907	0.942	0.983	0.951
DCNN variable-sized (test)	0.880	**0.988**	0.936	0.907	0.933	**0.985**	0.950
Proposed (test)	**0.977**	0.910	**0.982**	**0.979**	**0.943**	0.958	0.877

Bold numbers are the highest scores in this catergory.

**Figure 5:**
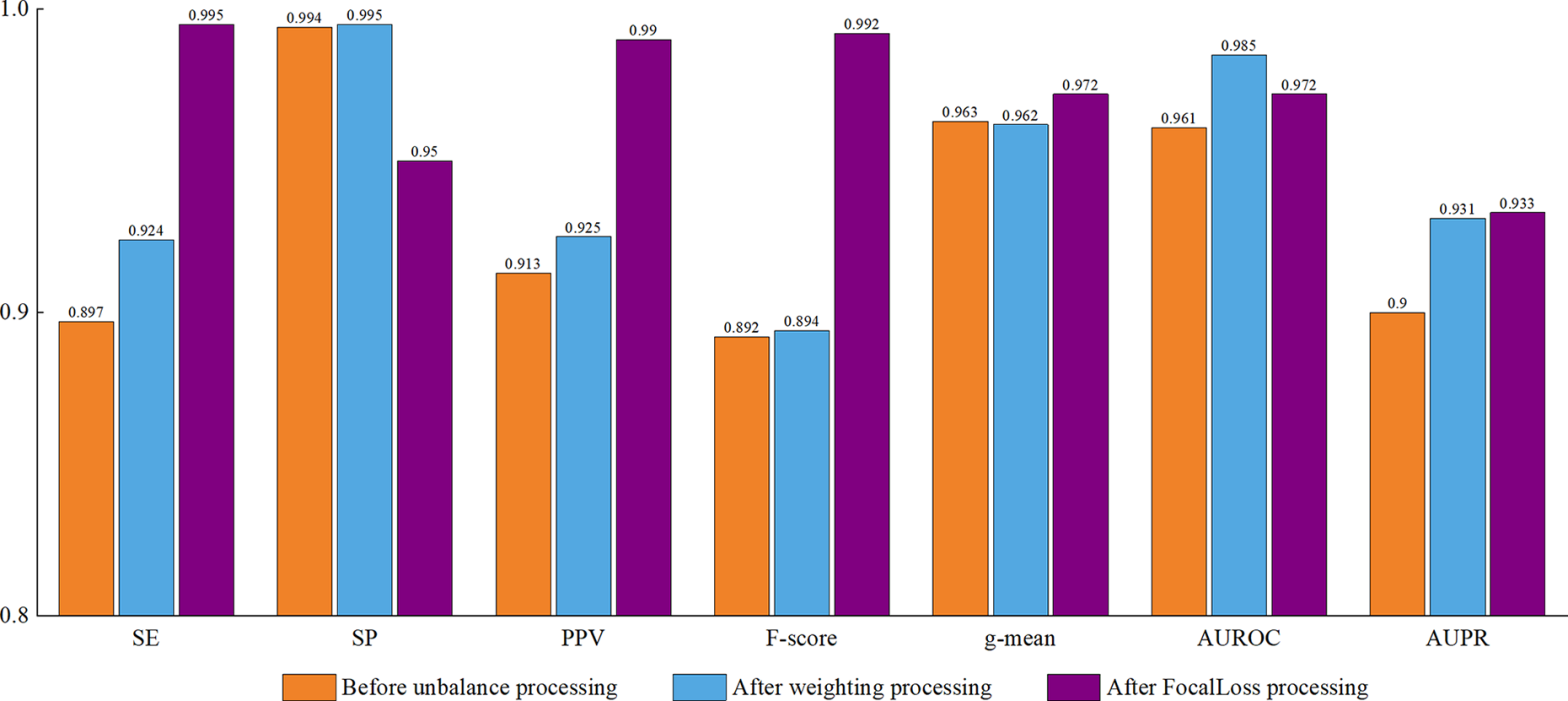
Comparison of prediction performance of our method and other comparison methods on cross-species datasets.

Our approach performed best overall in the case of the cross-species dataset. In the cross-validation, our method achieved the best values for SE, g-mean, F-score, and PPV; these were 10.19%, 3.4%, 10.47%, and 8.67% higher than the best results obtained by a comparison method, respectively, and our method ranks the second on AUPR. The reduction in SP occurred because training a classifier system with an unbalanced dataset (where the positive class is a minority) typically produces a suboptimal model with higher SP and SE (Batuwita and Palade, 2009a). In the test results, compared with DCNN, CL-PMI showed a 8.08% increase in SE. The performance with respect to the other metrics was the same in the cross-validation. This similarity indicated that overfitting had been effectively addressed. Some methods, such as miRBoost, showed fair performance in cross-validation, but poorer performance for each indicator with the test data. However, CL-PMI demonstrated the same level of performance in both cross-validation and testing, indicating that our approach has a more powerful generalization ability than the others. In order to more intuitively show the differences between the five methods for each indicator, we drew a radar chart, in which each indicator corresponded to a coordinate axis, and the relative position and angle of the axis were usually uninformed. **Figure 5** shows a comparison of the predicted performances for the cross-species dataset. We performed five-fold cross-validations and averaged the results. As illustrated by the radar chart, our method performed best on four of the seven indicators, and values for the remaining three were also close to optimal. This demonstrates that our proposed method is competitive in identifying pre-miRNAs.

**Figure 6:**
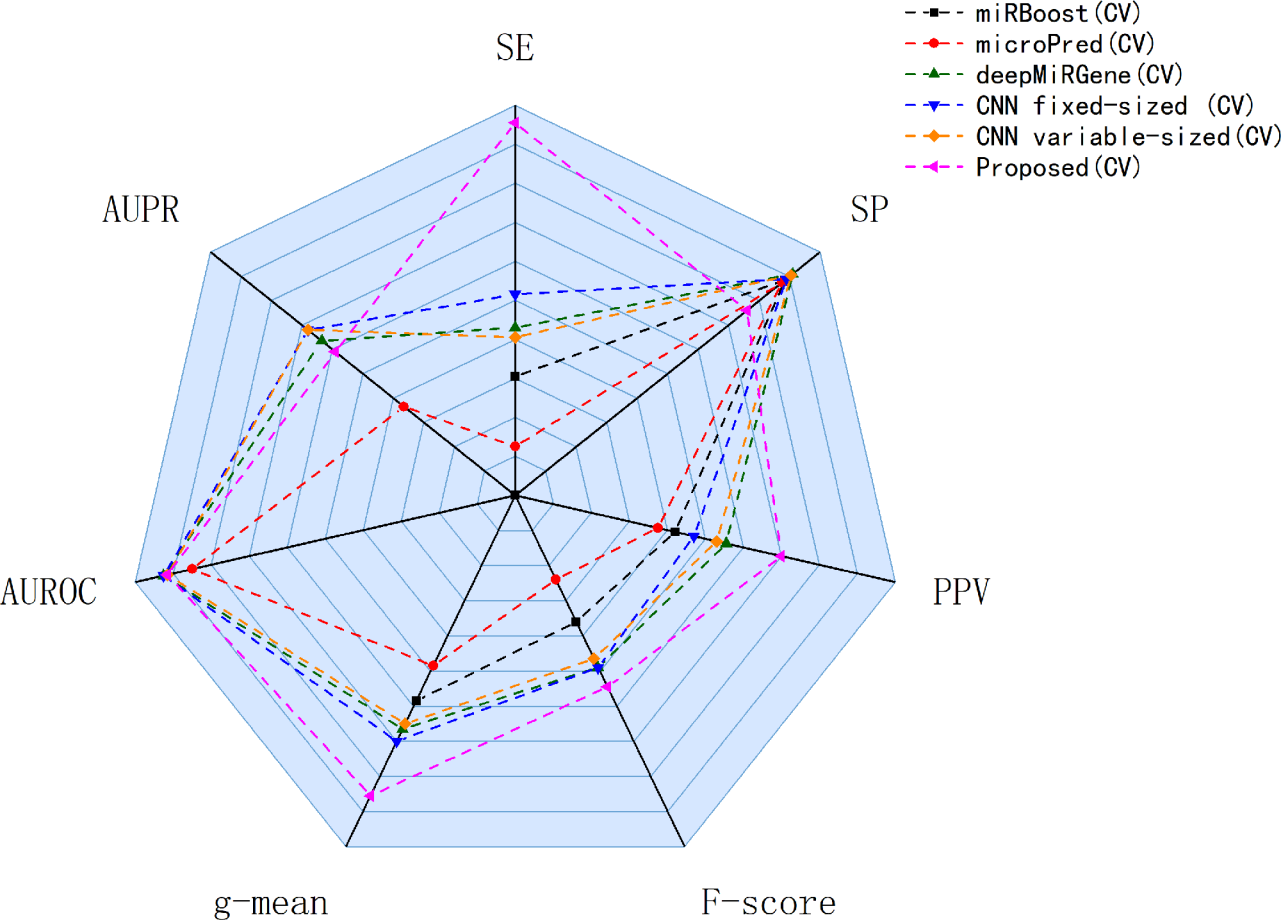
The AUROC curves of our method on the dataset human,cross-species, and new.

**Figure 7:**
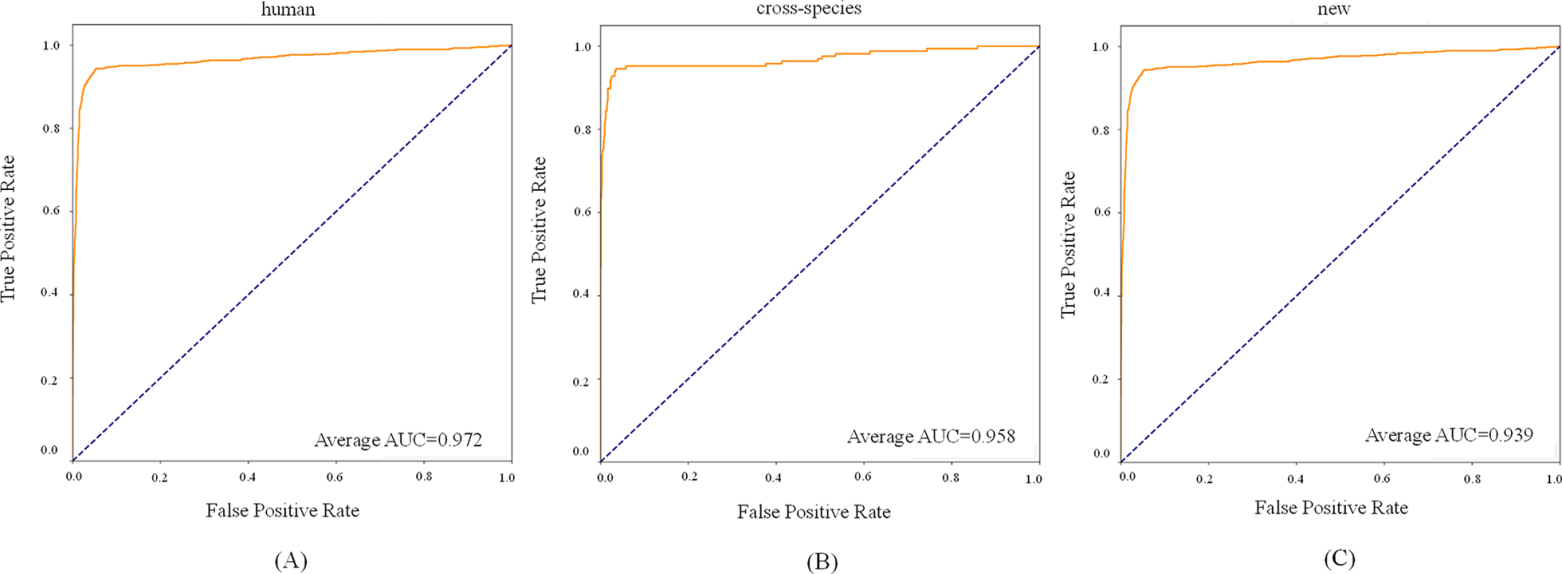
The AUROC curves of our method on the dataset human,cross-species, and new.

For the new dataset, in the basic indicators, CL-PMI showed a 5.32% increase in SE compared with miRBoost. In the comprehensive performance indicators, although our method was slightly worse than microPred in AUROC and AUPR.CL-PMI showed increases of 27.34% and 20.07% in PPV and F-score compared with DCNN, respectively. For a better view, we also plotted the AUC curves and AUPR curves of our method on human, cross-species, and new, respectively in **Figures 6** and **7**. We performed tests on the new dataset using the model trained on the cross-species dataset. The results are shown in **Table 4**. Although CL-PMI was trained on the mixed-species dataset, it showed potential for identifying new pre-miRNAs.

**Table 4:** Results for the new dataset.

Methods\metrics	SE	SP	PPV	F-score	g-mean	AUROC	AUPR
miRBoost	0.921	0.936	0.609	0.733	0.928	−	−
microPred	0.728	0.970	0.672	0.699	0.840	0.940	0.756
deepMiRGene	0.917	0.964	0.682	0.782	0.941	**0.981**	0.808
DCNN fixed-sized	0.917	0.967	0.696	0.792	**0.942**	0.979	**0.864**
DCNN variable-sized	0.859	**0.981**	0.779	0.817	0.918	0.979	0.818
Proposed	**0.970**	0.907	**0.992**	**0.981**	0.938	0.939	0.746

Bold numbers are the highest scores in this category.

For human datasets, according to the five-fold cross-validation results, CL-PMI outperformed other comparison methods with respect to SE, F-score, PPV, and g-mean. Notably, the F-score of our method was increased by 16.45% compared with that of DCNN; the other metrics also improved by 12.64%, 11.71%, and 3.89% compared with the best methods. For the test set, our method achieved the best performance in terms of SE, F-score, and PPV; although it did not give the highest scores on other metrics, the performance of CL-PMI was close to that of the best method.

We compared the performance of traditional machine learning and deep learning. In the human dataset, in addition to the highest SP achieved with microPred, the deep learning methods showed better performance for all metrics. For the cross-validation and testing of the cross-species dataset, the deep learning methods outperformed other methods. With the new dataset, the deep learning methods showed the best performance for all the evaluated metrics. All these results demonstrate that deep learning methods are superior to machine learning methods for identifying pre-miRNAs.

Our method achieved optimal results for SE, PPV, and F-score. Although deepMiRGene used LSTM to capture the long-term dependence of pre-miRNA, it did not focus on pre-miRNA spatial interaction, whereas DCNN used the CNN to focus on pre-miRNA spatial dependence but ignored complex space-time dependencies. CL-PMI considers both the sequential and spatial information of pre-miRNA, enabling the model to simultaneously express the characteristics of pre-miRNA in the spatial and time dimensions and thus achieve better classification results. The above results and analysis confirm that CL-PMI is competitive among deep learning methods. In addition, we tested our model on the new22 with an accuracy of 0.907. Related experimental details of new22 are shown in **Supplement C**.

### Impact of Class Imbalance on the Model

There was a certain degree of class imbalance in our experiments. That is, the number of positive examples (3,230 pre-miRNAs) was much smaller than that of negative examples (23,934 non-pre-miRNAs). The ratio of positive and negative examples was 1:7.4. Researchers have conducted extensive explorations of class imbalances. Minority classes are largely ignored and predicting them is more difficult, leading to degraded classifier performance (Weiss, 2004). To improve the model’s performance, it was necessary to solve the class imbalance problem.

In response to the dataset imbalance problem, we initially tried to use class weights, that is, we directly considered the asymmetry of the cost error during the classifier training, which embedded the output probability of each class in the cost error information. This probability was then used to define a classification rule with a 0.5 threshold. Specifically, the aim was to identify those small classes (positive pre-miRNAs) that could be used to add weight to the positive examples of the model and reduce the weight of the negative examples. This method produces a new data distribution, which allows the classifier to focus on positive examples. In this experiment, we set the positive example weight to 0.9 and the negative example weight to 0.1.

Later, we used the focal loss function, which is an elegant and effective proposal to solve the problem of class imbalance. In this function, γ is the focus parameter, which smoothly adjusts the reduced ratio of the weight of the easy sample, and (1-*p* *_y_*)^γ^ is the modulation factor, which reduces the loss contribution of the easy sample and broadens the range in which the sample receives low loss. When γ = 0, focal loss is equivalent to cross-entropy loss. When γ increases, the influence of the modulation factor increases accordingly. By adding the modulating factor, focal loss reduces the weight of the easy sample, making the model more focused on the hard sample during training.

**Figure 8:**
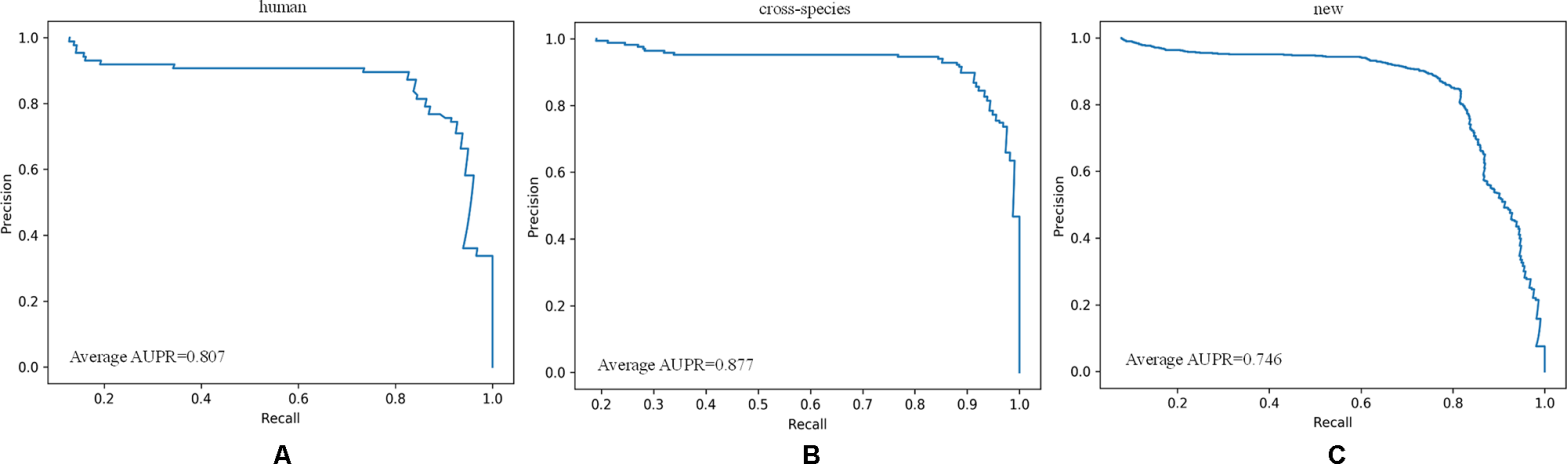
Performance assessment of cross-species dataset before and after unbalanced processing. The average score of the five-fold results is reported.

As shown in **Figure 8**, all of the performance metrics were higher after the class imbalance processing, except for g-mean and SP, although these showed small drops of only 0.001 and 0.042. The reduction of SP occurred because training a classifier system with an unbalanced dataset (where the positive class is a minority) typically produces a suboptimal model with higher SP and lower SE. By applying the class imbalance learning method, it is usually possible to increase the SE by sacrificing the SP score to some extent (Batuwita and Palade, 2009a). These results showed that our model was not biased towards the negative dataset. The focal loss method was superior to the class weight approach for dealing with imbalances. To be specific, the model achieved a 7.25% higher SE using focal loss compared with class weight. Similarly, for F-score, PPV, and g-mean, using focal loss resulted in 1.68%, 1.62%, and 0.94% higher scores compared with class weight. Therefore, we propose that focal loss is an effective means to deal with the class imbalance problem in pre-miRNA datasets.

## Conclusions

In this paper, we proposed a new pre-miRNA identification method, called CL-MPI. In contrast to existing methods, CL-MPI captures sequence information while also considering secondary structure in data preprocessing. CL-MPI can take into account pre-miRNA sequential and spatial information while automatically extracting pre-miRNA sequence features. We used RNAfold to predict the secondary structure of each pre-miRNA sequence, then used the secondary structure and sequence as inputs to a CNN to automatically extract features, and finally used LSTM to mimic RNA sequences and further understand the role of the sequence. According to the experimental results, our method achieved better overall performance across cross-species datasets, even in the absence of known manual features, especially for F-score and g-mean. This demonstrates that automatic extraction of features and considering sequential and spatial characteristics at the same time are important for the identification of pre-miRNAs. Owing to the small number of known pre-miRNAs, we needed to deal with extremely unbalanced datasets, with significantly more negative than positive examples. According to Saito and Rehmsmeier (2015), PPV is more useful than other metrics for a binary classifier on an unbalanced dataset, as it varies with positive and negative ratios. As described in the section *Results and Discussion*, we used 3,230 positive examples and 23,934 negative examples to train the CL-MPI model and obtained better performance for PPV compared with alternative methods. The higher SE and PPV achieved for the unbalanced dataset metrics prove that our method can better predict pre-miRNAs.

miRNAs are directly involved in tumor formation (Søkilde et al., 2014), which makes it possible to treat tumors using target carcinogenic miRNAs to restore the function of tumor suppressor miRNAs. With advances in clinical research, miRNAs continue to provide new ideas and treatments for tumor molecular diagnosis and treatment. Biomedical researchers are reluctant to use the outputs of “black box” methods as they are difficult to interpret, which affects the credibility of the results. Our next goal will be to explore more efficient visualization methods. In the future, we will also extend our method to other miRNA-related tasks, such as miRNA target prediction (Iqbal et al., 2016) and miRNA gene expression. Owing to the limited number of known miRNA sequences, the processing of unbalanced datasets also remains a challenge for future work.

## Data Availability Statement

All datasets analyzed for this study are included and cited in the manuscript and the **Supplementary Files**.

## Author Contributions

HW and YM performed the majority of the analysis and primarily wrote the manuscript. CD performed some analysis and provided biological expertise. CD performed some analysis of data and helped conceive the project. JW and DL completed the drawing of the charts in the results analysis and the layout of the manuscripts. All authors edited and approved the manuscript.

## Funding

This study was supported by research grants from the National key research and development plan of China (2018YFD0100204), the National Natural Science Foundation of China (61672374), and the Scientific and Technological Project of Shanxi Province (No.201603D22103-2).

## Conflict of Interest

The authors declare that the research was conducted in the absence of any commercial or financial relationships that could be construed as a potential conflict of interest.
