# Supplementary material for: CL-PMI: A Precursor MicroRNA Identification Method Based on Convolutional and Long Short-Term Memory Networks
Source: Front Genet. 2019 Oct 11;10:967. doi: 10.3389/fgene.2019.00967 (PMC6798641; doi:10.3389/fgene.2019.00967)
Supplement: Supplementary file 1 [file DataSheet_1.pdf]

Appendix

A hyperparameter tuning

Table A: Performance evaluation with varying numbers of filter dimension and LSTM units.

Filter dimension	LSTM unit	SE	SP	F-score	PPV	g-mean	AUROC	AUPR
20	20	0.980	0.964	0.989	0.990	**0.987**	**0.987**	0.910
20	30	**0.993**	0.954	**0.992**	0.991	0.973	0.974	**0.931**
20	40	0.991	0.954	0.991	0.991	0.972	0.972	0.922
30	20	0.983	0.948	0.986	0.990	0.966	0.966	0.882
30	30	0.963	**0.965**	0.967	0.977	0.953	0.966	0.891
30	40	0.982	0.964	0.988	**0.992**	0.973	0.973	0.891
40	20	0.988	0.939	0.988	0.988	0.964	0.964	0.896
40	30	0.984	0.960	0.988	**0.992**	0.972	0.972	0.894
40	40	0.988	0.957	0.989	0.991	0.972	0.972	0.908

Table B: Parameter settings for each layer in the model

Layer	Params
Convolutional (sequence)	$4 \times 40 \times 20$
Convolutional (secondary structure)	$3 \times 40 \times 20$
Maxpool (sequence)	2×2
Maxpool (secondary structure)	2×2
BLSTM	30
fully connected	256

Our proposed model is divided into convolution layer, pooling layer, LSTM layer and fully connected layer. The parameter details of each layer are set as shown in Table 6.

CNN and LSTM hyperparameter tuning: In order to find the optimal number of convolution kernels and LSTM units in our model, we set different configurations to evaluate its performance. As shown in Table 5, using 20 convolution kernels for the CNN layer and 30 units for the LSTM layer produced the best result. Using more units in either layer degraded the performance, probably due to overfitting. We tried more convolution kernels and units in CNN and LSTM, but the performance of the model is reduced, probably because of overfitting.

B Pseudocode of the Proposed Algorithm

Algorithm1 Pseudocode of our proposed methodology for pre-miRNA prediction

1: **Input:** $x_s \in \{A, U, C, \ \}^{|x_s|}$

▷x_s: a pre-miRNA sequence with its length of $|x_s|$.

2: Param: b: mini-batch size

3: Output: $y \in \{0,1\}$

▷ y: a true label for x_s.

Step 1: Encoding sequence and structure (Section2.1)

4: **for** each sequence x_s

5: $x_{ss} \leftarrow$ RNAfold (x_s)

6: ▷RNAfold: a tool to predict secondary structure of a given pre-miRNA sequence x_s.

▷x_{ss} : secondary structure of pre-miRNA sequence represented by dot bracket notation. $|x_{ss}|=|x_s|$ and $x_{ss} \in \{(,.,)\}$.

7: $X_s \leftarrow$ encode (x_s)

$X_{ss} \leftarrow$ encode (x_{ss})

▷ encode: a function that use one-hot encoding scheme to convert the nucleotides at each position of the pre-miRNA sequence into four-dimensional vectors, the observable state of each position of the secondary structure is converted into a three-dimensional vector.

▷X_s : a one—hot encoded matrix. $X_s \in \{0,1\}^{|x_s| \times 4}$.

▷X_{ss} : a one—hot encoded matrix. $X_{ss} \in \{0,1\}^{|x_{ss}| \times 3}$.

Step 2: Training on neural network (section 2.2 and 2.3)

8: initialize weights W

9: **for** each epoch

10: **for** b training data composed of X_s and X_{ss}, which is randomly picked from training samples

CNN Layer (section 2.2)

11: $C_s \leftarrow$ conv (X_s)

12: $C_{ss} \leftarrow$ conv (X_{ss})

13: ▷conv(): convolution layer to return feature map as an input of pooling layer.

14: ▷C_s: feature map of sequence after convolution operation.

15: ▷ C_{ss} : feature map of secondary structure after convolution operation.

16: $C_t \leftarrow$ pool(C_s)⁀pool(C_{ss})

17: ▷ pool(): pooling layer, which maximizes the output of each filter using max-pooling.

18: ▷⁀ : vector concatenate operation.

19: ▷C_t: final output vector of the CNN layers, which concatenate sequence and secondary structure.

LSTM Layer (section 2.3)

20: $L_t \leftarrow$ LSTM (C_t)

21: ▷LSTM: LSTM layer to return a vector for an input matrix.

22: ▷L_t: a 30-dim vector for the output of LSTM layer.

Fully Connected Layer

23: $y \leftarrow$ FC (L_t)

24: ▷FC: fully connected layer to return an output vector.

25: ▷ $y \in R^2$ (sigmoid activated).

26: ▷ y: an output of probability values in the range 0-1.

Weight Update (section 2.5)

27:E $\leftarrow -\frac{1}{b}\sum_i(\alpha(1-y_i)^{\gamma}\log y_i + (1-\alpha){y_i}^{\gamma}\log(1-y_i))$

28:▷E: mini-batch training error obtained using dynamically scaled cross entropy (focal loss).

29:▷ b: mini-batch size (b=256).

30:▷α ∈[0,1] is a weighting factor corresponds to class 1, and 1-α corresponds to class -1.

31:▷ γ: focus parameter.

32:W $\leftarrow$ W $-\Delta$W

33:▷calculate ΔW based on E using gradient descent optimization algorithm "Adam".

C Results for the new22 dataset

Table C: Performance of our model in the new22 dataset

SE	SP	F-score	PPV	g-mean	AUROC	AUPR	ACC
0.874	0.869	0.787	0.778	0.846	0.847	0.876	0.907

We obtained positive samples from miRBase22, and obtained negative examples from Xue and Zou as new22 dataset. To avoid overfitting, we remove the sequences that have an identity of >97% with the other ones using CD-HIT(Li & Godzik, 2006). Finally, we selected 690 positive examples and 8246 negative examples. The result of new22 is shown in Table 7.

References

Li, W., & Godzik, A. (2006). Cd-hit: a fast program for clustering and comparing large sets of protein or nucleotide sequences. *Bioinformatics*, *22*(13), 1658.
